# The Antimicrobial Properties of Chitosan Can Be Tailored by Formulation

**DOI:** 10.3390/md18020096

**Published:** 2020-01-31

**Authors:** May Wenche Jøraholmen, Abhilasha Bhargava, Kjersti Julin, Mona Johannessen, Nataša Škalko-Basnet

**Affiliations:** 1 Drug Transport and Delivery Research Group, Department of Pharmacy, Faculty of Health Sciences, University of Tromsø The Arctic University of Norway, Universitetsveien 57, 9037 Tromsø, Norway; may.w.joraholmen@uit.no (M.W.J.); abhilasha.b-94@hotmail.com (A.B.); 2 Research group for Host-Microbe Interaction, Department of Medical Biology, Faculty of Health Sciences, University of Tromsø The Arctic University of Norway, Sykehusveien 44, 9037 Tromsø, Norway; kjersti.julin@uit.no (K.J.); mona.johannessen@uit.no (M.J.)

**Keywords:** chitosan-coated liposomes, chitosan hydrogel, mucoadhesion, vaginal infections, antibacterial activity, *Staphylococcus epidermidis*, *Staphylococcus aureus*

## Abstract

Topical administration of drugs into the vagina can provide local therapy of vaginal infections, preventing the possible systemic side effects of the drugs. The natural polysaccharide chitosan is known for its excellent mucoadhesive properties, safety profile, and antibacterial effects, and thus it can be utilized in improving localized vaginal therapy by prolonging the residence time of a drug at the vaginal site while acting as an antimicrobial in synergy. Therefore, we aimed to explore the potential of chitosan, namely chitosan-coated liposomes and chitosan hydrogel, as an excipient with intrinsic antimicrobial properties. Liposomes were prepared by the thin-film hydration method followed by vesicle size reduction by sonication to the desired size, approximately 200 nm, and coated with chitosan (0.01, 0.03, 0.1, and 0.3%, w/v, respectively). The mucoadhesive properties of chitosan-coated liposomes were determined through their binding efficiency to mucin compared to non-coated liposomes. Non-coated liposomal suspensions were incorporated in chitosan hydrogels forming the liposomes-in-hydrogel formulations, which were further assessed for their texture properties in the presence of biological fluid simulants. The antibacterial effect of chitosan-coated liposomes (0.03%, 0.1% and 0.3%, w/v) and chitosan hydrogels (0.1% and 0.3%, w/w) on *Staphylococcus epidermidis* and *Staphylococcus aureus* was successfully confirmed.

## 1. Introduction

Although the antibiotics era enabled treatment of previously fatal infections, microorganisms managed to “fight back” and develop resistance, leading to an era of antimicrobial resistance. As a consequence, the antimicrobial treatment options became limited and the need for better antimicrobials more evident. In a search for novel antimicrobials, materials of natural origin with intrinsic antimicrobial properties become highly attractive, especially for localized antimicrobial therapy. A material exhibiting intrinsic antimicrobial properties can be used either as a pharmaceutical excipient, for example, as a vehicle for the antimicrobial agent, or as an active agent itself [1]. The choice of excipients of natural origin with intrinsic antimicrobial properties will be dependent both on the targeted microorganism but also on the features of the administration site such as skin, vagina, etc. The choice will also be influenced by the other characteristics of the material such as its muco- and bio-adhesiveness, stability in biological environment, toxicity, etc. Considering the vagina as an administration site, chitosan is among the most promising materials. We have extensively studied chitosan-based delivery systems [2,3,4,5], both for skin and vaginal administration. To date, no consensus in the field has been reached considering the exact mechanisms of the antimicrobial actions of chitosan. The antimicrobial effects of chitosan are attributed to its ability to destabilize the outer membrane of Gram-negative bacteria [6,7] and permeate the microbial plasma membrane [8]. The interaction between positively charged chitosan molecules and negatively charged microbial cell membranes is expected to lead to a disruption of microbial membrane, followed by a leakage of intracellular constituents [9]. It was proposed that at a lower concentration (< 0.2 mg/mL), the cationic groups of chitosan bind to the negatively charged bacterial surface leading to agglutination, while, at higher concentrations, the larger number of chitosan cationic groups form a net positive charge onto the bacterial surfaces resulting in a suspension [6]. Considering the optimal properties of chitosan, it seems that its hydrophilicity is essential for its antimicrobial potential. In addition, its molecular weight, degree of acetylation and ionic strength and pH of the dissolving medium will also affect antimicrobial properties of chitosan. Therefore, by tailoring the formulation features, it is possible to optimize the antimicrobial potential of chitosan-based formulations [10].

Genital infections can be caused by a variety of microorganisms. However, bacterial vaginosis remains among the most recurrent infections of genital tract [5]. There are several factors responsible for failure to eradicate bacterial vaginosis completely and prevent recurrence. However, it seems that persistent bacterial biofilms could be among the most contributing factors. Antibiotics fail to fully penetrate the negatively charged polysaccharide matrix coating the bacteria in biofilm, enabling the survival of bacteria in the deeper quarters of the biofilm. Therefore, utilizing material able to act on disruption of biofilms, as well as deliver other antimicrobial of interest within the same formulation, may lead to successful antibacterial therapy. Chitosan was proposed as a potent antimicrobial material acting on biofilms; chitosan gels were reportedly able to eradicate *Pseudomonas aeruginosa* biofilms in a pH-independent manner. Moreover, the chitosan concentration required to eradicate biofilms was rather low (0.13%) [11].

Treating vaginal infections requires careful tailoring of the formulation features, since the vagina as an administration site bears specific challenges which should be addressed/overcome when optimizing the therapy. The first consideration is probably the need to assure that the formulation does not disturb the natural vaginal environment [12]. A further challenge is sufficient residence time within the vaginal cavity. Formulations such as liposomes-in-hydrogel formulations can assure the required mucoadhesive properties and vaginal residence time [13,14,15]. A liposomes-in-hydrogel formulation can exhibit a synergic effect; poorly soluble active substances/drugs will be incorporated in liposomes whereas the extend residence time within the vaginal site will be assured by hydrogel as a vehicle [16,17]. Moreover, these hydrogels are often based on natural mucoadhesive polymers such as chitosan, assuring the formulation’s biocompatibility and biodegradability [18].

In order to achieve maximal clinical outcome of novel formulation based on hydrogels in terms of improving retention of a drug and spreading within the vaginal cavity, it is necessary to highlight the importance of texture characterization of hydrogels [19]. Utilizing chitosan as a hydrogel vehicle with intrinsic biological activity enables synergy between the drug and excipient chitosan [20].

The additional advantage of chitosan is its ability to closely interact with mucus, thus providing an efficient contact-time between the formulation and the vaginal mucosal epithelium [3]. Chitosan can be used to prepare different mucoadhesive delivery systems and dosage forms either as a coating material for liposomes and a building block for nanoparticles or as a mucoadhesive hydrogel [21,22].

We aimed to evaluate whether the formulation type and features have an impact on the antimicrobial performance of chitosan-based formulations. To avoid interference from the active ingredients, we focused on drug-free formulations. We prepared and fully characterized two main formulation types, namely chitosan-coated liposomes and liposomes-in-chitosan hydrogel. The formulations were fully characterized and tested against *Staphylococcus epidermidis* and *Staphylococcus aureus* and their antimicrobial activity compared.

## 2. Results and Discussion

An optimal localized treatment of vaginal infections depends not only on the potency of the active ingredient/drug, but also on the physiochemical properties of the formulation; an ideal formulation can protect and enhance as well as act in synergy with antimicrobial to assure successful therapy [23]. Chitosan-based delivery systems exhibit strong mucoadhesive properties, an excellent safety profile and intrinsic antimicrobial activity of chitosan, which add to their attractiveness as pharmaceutical formulations, including those destined for vaginal delivery [4,17,24,25]. However, relatively little is known about the effects of the type of formulation on the antimicrobial performance of the formulation. Since the most interesting chitosan-based formulations are coated liposomes and chitosan hydrogels, we developed, characterized and evaluated these two formulations.

### 2.1. Liposomal Characteristics

The vesicle size of liposomes depends on the preparation method, and the thin-film hydration method is known to generate rather large heterogeneous multilamellar vesicles (MLVs). [26]. When aiming at vaginal mucosal delivery, the vesicle size range is suggested to be approximately 200 nm [12]. We, therefore, reduced the MLVs’ size to close to 200 nm (Table 1). The size reduction by probe sonication resulted in a bimodal size distribution expressed as two vesicle populations. However, the polydispersity index (PI) was found to be acceptable with values below 0.4 (Table 1). A lower PI value indicates a more homogenous liposomal distribution [27].

An increase in liposomal size was seen for the chitosan-coated liposomes coated with the higher chitosan concentration (0.1% and 0.3%, w/v), indicating that coating was successful. Further, the vesicle size increased with the increasing polymer concentration, in agreement with the literature [3,28,29,30,31]. Although the chitosan concentrations commonly utilized for coating of vesicles range from 0.1% to 0.6%, we tried to use even lower concentrations (0.01% and 0.03%, respectively). However, the lowest concentration of chitosan did not lead to an increase in the original liposomal size (Table 1). Lack of the change in vesicle size after the coating may indicate that the coating was unsuccessful, assuming that original vesicle size of neutral liposomes was representative. The polydispersity index indicates a rather heterogenous population of neutral liposomes (Table 1). NICOMP distributions categorize the vesicles in subpopulations of vesicles of similar size [28]. Therefore, results are an estimate based on the intensity of subpopulations. In this case, the degree of significance would not be relevant and was not calculated.

**Figure 1:**
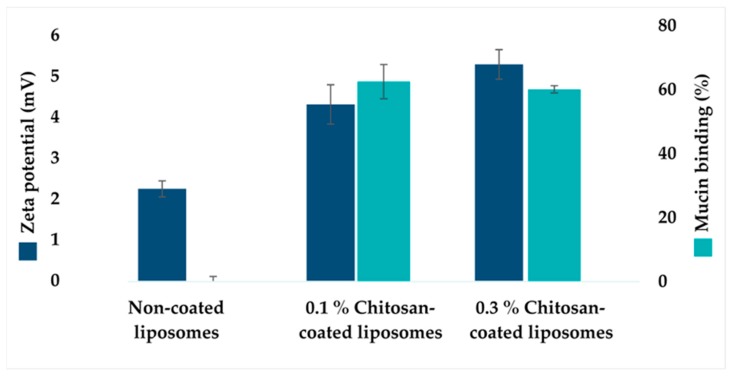
The zeta potential of chitosan-coated liposomes and effect of chitosan coating on mucin binding. In dark blue: The effect of chitosan coating on the zeta potential of liposomes. The values denote the mean of three individual experiments, determined in triplicates, and expressed as the mean ± SD for non-coated liposomes, 0.1 and 0.3% chitosan-coated liposomes, respectively. In light blue: The effect of chitosan coating on the mucin binding efficiency. The values are presented as the mean ± SD (n = 3).

An increased zeta potential for chitosan-coated liposomes is expected due to the cationic character of chitosan and can be used as an indicator of successful coating. The zeta potential of all liposomal formulations was determined (Figure 1), confirming that chitosan coating with the higher chitosan concentration (0.1% and 0.3% w/v) resulted in an increase in liposomal surface charge compared to the non-coated liposomes, in agreement with the literature [3,32]. The increase in potential was lower compared to our previous findings [28,29]. However, the use of different origin chitosan, lipid, and the medium liposomes were dispersed in, might be the contributing factors to the observed differences [33]. The changes in the liposomal size (Table 1) and increase in zeta potential (Figure 1) confirmed the successful coating when higher chitosan concentrations were applied.

To prolong the residence time of vaginal formulation, it is advantageous to increase the concentration of polymer thereof, enhancing the strength of mucoadhesion [34]. The mucoadhesive properties of chitosan-coated liposomes were determined based on the mucin binding of chitosan-coated liposomes compared to non-coated liposomes (Figure 1) [29]. The mucin binding of chitosan-coated liposomes was significantly higher (p < 0.001) than non-coated liposomes, as expected. Results corresponded to earlier findings regarding increased mucin binding due to chitosan coating [28,29,35]. However, the 0.3% (w/v) chitosan concentration did not express significantly increased mucin binding compared to 0.1% (w/v) chitosan-coated liposomes. In our previous work [29], we determined the surface availability of chitosan when two different concentrations of chitosan were used to coat liposomes. The availability of chitosan was higher when the coating was performed with lower chitosan concentrations. The mucin-binding was also superior. It is important to consider that these findings can only provide indirect information about the binding efficiency of chitosan coating on the liposomal surface. The difference between the current and previous work can be contributed to chitosan origin, different molecular weights of chitosan, as well as different size of liposomes that were coated. The longer polymer chains are more likely to interact to a lesser extent with mucin chains, resulting in reduced interpenetration through vaginal mucus, and thus reduced adhesive properties [3].

### 2.2. Hydrogel Characteristics

Chitosan-based hydrogels are considered to be very attractive vehicles for vaginal drug delivery due to their lower pH (4–5), high water content, biodegradability and pronounced mucoadhesiveness [24].

The texture properties of hydrogels, such as the hardness, cohesiveness and adhesiveness, are essential parameters to be evaluated when considering hydrogel as a potential vaginal formulation. In brief, the hardness of the hydrogel indicates the applicability of the hydrogel, considering the ease of application, packaging and storage. The adhesiveness may indicate the contact time between the hydrogel and mucus and therefore the retention within the vaginal cavity. Cohesiveness describes the work required to deform the hydrogel in the downward movement of the probe [36]. The composition of the hydrogels determines their textural properties, which can be further assessed as an indicator to obtain optimal properties suitable for the specific route of administration. Thus, these parameters were investigated for various types of hydrogel formulations to assure that the properties of hydrogels correlate to the desired features. These properties of hydrogels are known to be influenced by the molecular weight of the polymer, and previous findings indicated medium-molecular-weight chitosan to exhibit superior texture properties, considering vaginal administration, compared to the high and low-molecular-weight chitosan [17]. Thus, hydrogels based on medium-molecular-weight chitosan were used in all experiments in this study.

**Figure 2:**
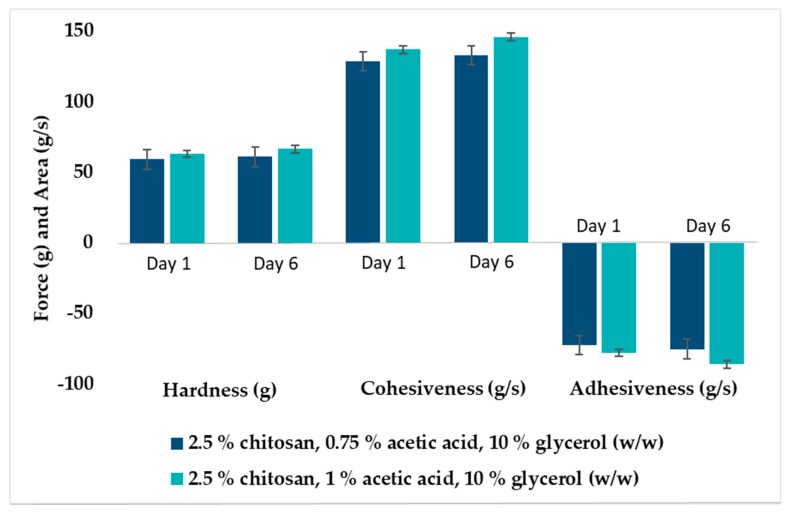
Texture properties of chitosan hydrogels (2.5% w/w) with different compositions determined either as freshly prepared or after storage for 5 days at room temperature (23–25 °C). The values are expressed as the mean ± SD (n = 3).

Glycerol was added to hydrogels to maintain the stability of chitosan hydrogels [37]; moreover, glycerol has also shown the ability to improve the texture properties of the hydrogels [17,36]. To avoid possible toxicity and local irritation, we aimed to minimize the acetic acid concentration used in hydrogel preparation. Hydrogels with 0.75 and 1% (w/w) acetic acid were prepared to investigate the effect of acid concentration on texture properties and short time stability. A slight increase in the hardness, cohesiveness and adhesiveness was seen for hydrogels made with 1% (w/w) acetic acid after storage for 5 days at room temperature (Figure 2). The same trend was observed previously [17] for hydrogels stored for 2 months. This was not observed for hydrogels made with a lower concentration (0.75%) of acetic acid (Figure 2). Moreover, the reproducibility of hydrogels comprising 1% (w/w) acetic acid was superior to 0.75% (w/w). This might indicate that 0.75% (w/w) acetic acid is the minimum acid concentration needed to assure chitosan solubility. Additionally, all parameters were increased for the hydrogels comprising an increased concentration of acetic acid, possibly due to enhanced interactions between chitosan and acetic acid [38]. Hence, acetic acid with a concentration of 1% (w/w) was used for the preparation of hydrogels in all further experiments. We did not encounter any toxicity issues for hydrogels prepared with even higher chitosan concentrations (data not shown).

### 2.3. Effect of Biological Fluids on The Texture Properties of Chitosan Hydrogels

All formulations administered to the vaginal cavity will be exposed to biological fluids which may affect their properties and consequently their performance [17]. The composition, volume and pH of vaginal fluids varies with women’s age and stage in reproductive cycle [39]. The mucoadhesive vaginal drug delivery system can be optimized by utilizing information on texture properties provided by the texture analysis [17,19]. To explore the influence of relevant biological fluids on the texture properties of hydrogels, the hardness, cohesiveness and adhesiveness of hydrogels were determined when the hydrogels were exposed to mucin, vaginal fluid simulant (VFS) and human semen fluid simulant (SFS).

**Figure 3:**
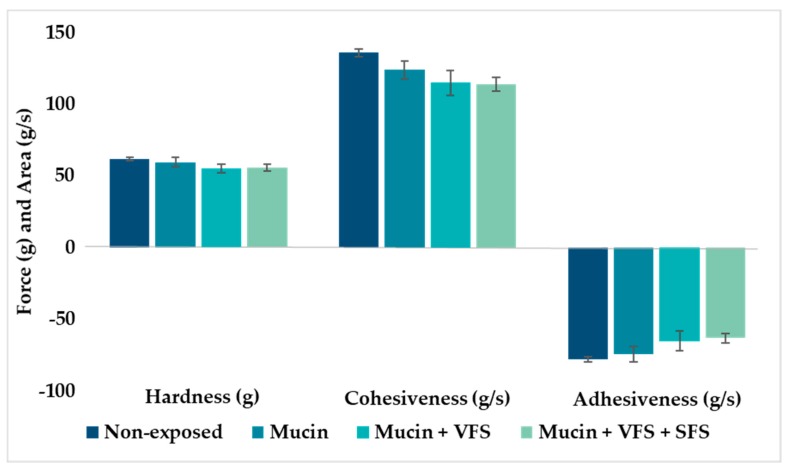
Texture properties of hydrogels (2.5% w/w chitosan) in the presence of mucin, vaginal fluid simulant (VFS) and semen fluid simulant (SFS) as compared to non-exposed hydrogel. The values are shown as the mean ± SD (n = 4).

Pig mucin was chosen as a model mucin due to its similarity to human mucin [40]. The cohesiveness and adhesiveness of the chitosan hydrogel was significantly reduced (p < 0.001) in the presence of mucin, compared to the non-exposed hydrogels, indicating that mucin is affecting the texture properties (Figure 3). Theoretically, adhesiveness should not be significantly affected due to enhanced adhesion between the polymeric hydrogel and mucin [41]. The hydrogel hardness was not affected by the presence of mucin. However, when hydrogels were exposed to the mixture of biological fluids, all texture parameters were affected and reduced as compared to intact hydrogels. The texture properties of hydrogel in the presence of vaginal fluids may indicate the level of robustness and viscosity of the hydrogel, which can affect the spreadability of the hydrogel and optimal coverage of vaginal mucus. The introduction of vaginal and semen fluid will alter both the viscosity and pH of vaginal mucus, and possibly contribute to improved ability of the hydrogel to spread evenly onto the vaginal mucus, increasing the contact time between mucin and the mucoadhesive hydrogel. However, the strength of the interactions between mucin and hydrogels might be weakened [12]. Similar trends as observed in our testing have been reported for both poloxamer and chitosan-based hydrogels [42,43].

Many of the active ingredients/drugs destined for localized vaginal therapy are poorly soluble and cannot be dissolved in hydrogels, therefore requiring a carrier able to solubilize them, as well as offer protection against the hydrogel microenvironment [23]. Liposomes are among the most studied carriers for poorly soluble substances/drugs. Due to their liquid nature, liposomes often require secondary vehicles such as a hydrogel to assure prolonged residence time within the vaginal cavity. The incorporation of liposomes in hydrogels has been shown to improve formulation texture properties [17]. Liposomes not only enable the incorporation of poorly soluble drugs, but also act on improved bioavailability and potential controlled drug release. The combination of liposomes and chitosan hydrogel, liposomes-in-hydrogel formulation, provides a prolonged residence time at the vaginal site and improved localized drug therapy. The incorporation of liposomes in hydrogel has also been shown to increase their stability when exposed to vaginal fluids [44]. Hence, we tested how biological fluids influence the texture properties of liposomes-in-hydrogel formulation.

**Figure 4:**
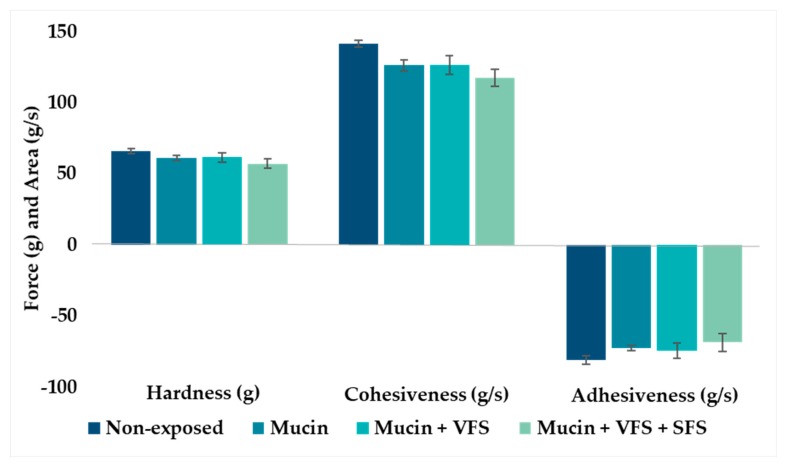
Texture properties of liposomes-in-hydrogels in the presence of mucin, vaginal fluid simulant (VFS) and semen fluid simulant (SFS). The values are shown as the mean ± SD (n = 3).

The incorporation of liposomes into hydrogels resulted in hydrogels exhibiting slightly increased adhesiveness and cohesiveness (Figure 4) compared to hydrogels containing buffer in the same concentration as the liposomal suspension (Figure 3). This finding closely corresponded to earlier findings, indicating that liposomes are indeed stabilizing the chitosan network [36]. However, the texture properties of liposomes-in-hydrogel formulation were also affected by the exposure to biological fluids (Figure 4).

### 2.4. Antimicrobial Effects of Chitosan Formulations

After optimizing the two chitosan-based formulations, we evaluated the effect of formulation features on the antimicrobial activity. The antimicrobial properties of chitosan are widely studied [1,7,45]. To characterize the antibacterial activity, we opted to compare two of the most common chitosan-based formulations, namely chitosan-coated liposomes versus chitosan-based hydrogels. The antibacterial effects of chitosan-coated liposomes (0.01%, 0.03%, 0.1% and 0.3%, w/v respectively) and chitosan hydrogel (0.1% and 0.3% w/w, respectively) were tested against methicillin-resistant or sensitive strains of two clinical species of Gram-positive bacteria; namely *S. aureus* and *S. epidermidis*. *Staphylococcus spp*. is naturally found on the human skin and mucosal surfaces. A disturbance in the microbiota can result in *Staphylococcus spp* infections, and poor hygiene, elevated pH, immune deficiency and diabetes can lead to extensive colonization of this bacteria [46]. Few studies have connected *Staphylococcus* to the vaginal environment; however, it is known that *Staphylococci* may contribute to bacterial vaginosis and increase the diversity of bacteria in vaginal microbiota [47]; hence, we chose to use these bacterial species as model organisms to evaluate the effect of the type of chitosan formulation on its antimicrobial properties.

**Figure 5:**
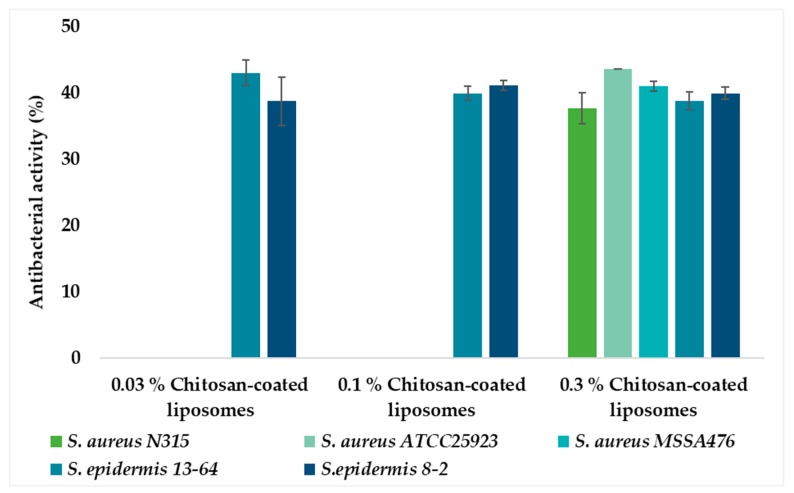
Antibacterial activity of chitosan-coated (%, w/v) liposomes expressed as the percentage of antibacterial activity compared to positive control vancomycin (100%). The values are presented as the mean ± SD (n = 3).

Non-coated (chitosan-free) liposomes were considered as a negative control and, as expected, did not show any antibacterial effect (data not shown). The neutral liposomal membrane is expected to have limited interaction with the bacterial cell membrane, thus, resulting in negligible antibacterial activity, as observed. The antibacterial activities of all liposomal formulations were expressed as a percentage of inhibition compared to a positive control; antibiotic vancomycin. Thus, the antibacterial activity of each formulation was expressed as a percentage of growth inhibition zone relative to the inhibition effect of vancomycin considered to be 100%. No bacterial growth inhibition was observed for liposomes coated with 0.01% chitosan for all bacteria isolates (data not shown) as expected. Interestingly, liposomes coated with chitosan concentration as low as 0.03% did suppress bacteria growth of *S. epidermidis*, whereas the same liposomes did not show the antibacterial effect on *S. aureus* (Figure 5). Those liposomes were very similar in size and zeta potential to non-coated liposomes and were expected to have a very thin layer of coating (Table 1). However, it seems that the available concentration of chitosan on the liposomal surface was sufficient to act as an antimicrobial.

To explain why the same liposomes acted on *S. epidermidis* but not on *S. aureus*, more work should have been included. However, it was shown early on that *S. aureus* is more prone to developing resistance than *S. epidermidis* [48], which could be among the contributing factors for the observed difference. The postulation may be supported by the fact that only the 0.3% chitosan-coated liposomes efficiently inhibited bacterial growth of *S. aureus*, indicating that the growth inhibition potential might be dependent on the chitosan concentration available on the liposomal surface. However, these differences were not statistically significant; and further evaluation of higher concentrations of chitosan-coated liposomes is needed to investigate the hypothesis of increased antibacterial effect at higher concentrations as well as broader antimicrobial spectra when higher chitosan concentrations are used for coating. An additional factor which may be interesting to explore is the size of liposomes. Liposomes coated with the highest chitosan concentration were larger in size than smaller vesicles (Table 1) and more mucoadhesive (Figure 1), which might imply that they were in closer contact with agar.

**Figure 6:**
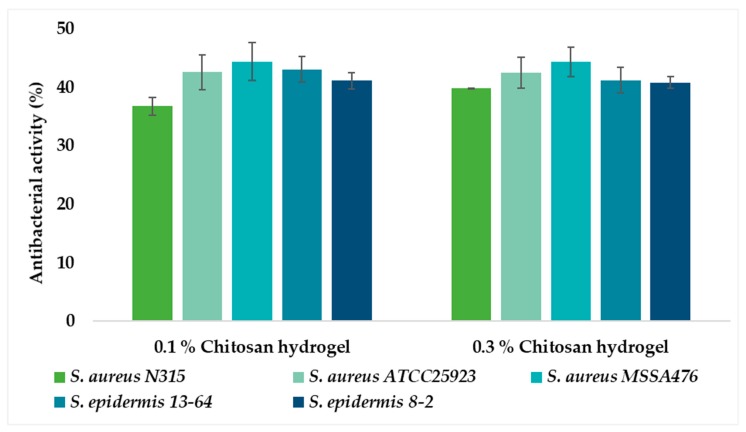
Antibacterial activity of chitosan hydrogels (%, w/w) expressed as the percentage of antibacterial activity compared to positive control vancomycin (100%). The values are presented as the mean ± SD (n = 3).

**Figure 7:**
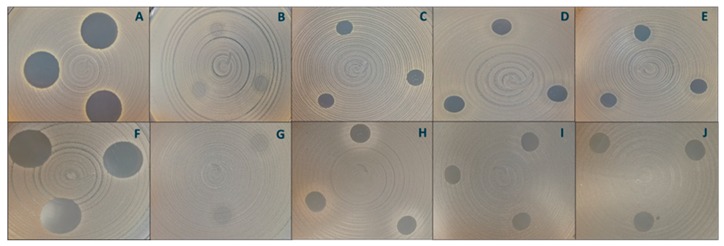
Antibacterial activity of chitosan formulations. Inhibition of *S. aureus MSSA 476* by vancomycin (**A**, positive control), non-coated liposomes (**B**, negative control), 0.3% (w/v) chitosan-coated liposomes (**C**), 0.1% (w/w) chitosan hydrogel (**D**) and 0.3% (w/w) chitosan hydrogel (**E**). Inhibition of *S. aureus ATCC 25923* by vancomycin (**F**, positive control), non-coated liposomes (**G**, negative control), 0.3% (w/v) chitosan-coated liposomes (**H**), 0.1% (w/w) chitosan hydrogel (I) and 0.3% (w/w) chitosan hydrogel (**J**).

No significant difference in antibacterial activity was observed between the two concentrations of chitosan hydrogels (0.1% and 0.3%, respectively), indicating that the lower chitosan concentration is sufficient to express an inhibitory effect against both bacterial strains (Figure 6). Hydrogels with 0.1% and 0.3% chitosan were proven to be more effective against *S. aureus* than the chitosan-coated liposomes (Figure 7). Possibly, less chitosan is available on the surface of the chitosan-coated liposomes which are spherical in nature, resulting in reduced electrostatic interactions between chitosan and the negatively charged bacterial cell membrane [26]. Recently, Dumont and colleagues [49] reported that alginate fibers coated with chitosan significantly decreased the bacterial growth of *S. aureus*. Moreover, they were able to confirm that formulation features also play an important role in antimicrobial activity of formulation; longer fibers were found to be better. Although their aim was to develop novel wound dressing, the findings are relevant for our study. It is interesting that the 0.1% chitosan-coated liposomes did not inhibit the growth of *S. aureus* to the same extent as 0.1% chitosan hydrogel, indicating that the type of formulation contributes to the degree of inhibition of this bacterium.

The zones of inhibition were from 0.00 cm for non-coated liposomes to 1.81 cm for the positive control, vancomycin.

Chitosan can be used as an excipient able to contribute as an antibacterial agent, as suggested by Yang and colleagues [20]. Previous studies have reported that the antimicrobial activity of chitosan depends on parameters such as molecular weight, degree of deacetylation and derivatization [50]. We have shown that the type of chitosan formulation can contribute to the overall antimicrobial performance of the formulation. These promising findings need to be further explored to confirm that the type of formulation affects not only the antibacterial but also antifungal activity of chitosan. Moreover, it would be interesting to evaluate the potential of chitosan derivatives as well as chitosan hybrid nanoparticles. For example, a quaternized chitosan was recently reported to exhibit strong activity against *Escherichia coli* when formulated as a nanofiber membrane [51]. Similarly, hybridization of chitosan with protamine lead to improved activity of chitosan nanoparticles against *E. coli* [52].

## 3. Materials and Methods

### 3.1. Materials

Lipoid S 80 (80% phosphatidylcholine from egg) was a gift from Lipoid GmbH, Ludwigshafen, Germany. Chitosan (medium-molecular-weight hydramer HCMF) was a gift from Chitinor AS, Tromsø, Norway. Mucin from porcine stomach (type III, bound sialic acid 0.5%–1.5%, partially purified), acetic acid, ammonium acetate, bovine serum albumin, calcium chloride, calcium hydroxide, fructose, glucose, glycerol, lactic acid, magnesium chloride, potassium chloride, potassium phosphate, sodium chloride, sodium phosphate dibasic, sodium phosphate monobasic monohydrate and vancomycin hydrochloride were all purchased from Sigma Aldrich Chemie GmbH, Steinheim, Germany. Potassium hydroxide and sodium citrate dehydrate were the products of NMD, Oslo, Norway. Urea was the product of Apotekproduksjonen AS, Oslo, Norway. *Staphylococcus aureus* MSSA476 (ATCC^®^ BAA-1721™) was purchased from LGC standard AB, Sweden. *Staphylococcus aureus* N315 was a gift from T. Ito (https://www.ncbi.nlm.nih.gov/pubmed/10348769). *Staphylococcus epidermidis* 8-2 was from Rikshospitalet, The University Hospital, Oslo, Norway, while *Staphylococcus epidermidis* 13-67 and *S. aureus* ATCC25923 were from University Hospital Northern Norway, Tromsø, Norway.

### 3.2. Preparation of Liposomes

Liposomes were prepared by the conventional thin-film lipid hydration method [29]. Phosphatidylcholine (1 g) was dissolved in excess methanol in a round-bottomed flask. The solvent was evaporated using a rotoevaporator (Büchi rotavapor R-124 with vacuum controller B-721, Büchi Vac^®^ V-500, Büchi Labortechnik, Flawil, Switzerland) for at least 1 h at 60 mBar and 45 °C, forming a thin-film lipid. The lipid film was dislodged from the flask walls by adding 50 mL acetate buffer (pH 4.6, 77.1 g/L CH_3_COONH_4_, 70 mL glacial acetic acid). The liposomal suspension was kept in a refrigerator (4–6 °C) overnight prior to further experiments.

### 3.3. Vesicle Size Reduction

The vesicle size of the liposomes was reduced by probe sonication [28]. The liposomal suspension was placed on ice and the needle probe tip inserted approximately 5–7 mm into the liposomal suspension. The sonicator (Ultrasonic processor 500 W, Sigma–Aldrich, St. Louis, MO, USA) was set to 40% amplitude and the samples sonicated 3 times for 1 min, with 1 min resting periods. The samples were stored in the refrigerator (4–6 °C) for at least 6 h prior to further experiments.

### 3.4. Chitosan Coating of Liposomes

The liposomes were coated with 0.01%, 0.03%, 0.1% and 0.3% (w/v) chitosan solutions, respectively. All solutions were prepared in 0.1% (v/v) glacial acetic acid. The chitosan solution (2 mL) was added drop wise into the liposomal suspension (2 mL) under magnetic stirring at a constant rate, at room temperature for 1 h [29]. Prior to characterization, the chitosan-coated liposomes were stored in a refrigerator (4–6 °C).

### 3.5. Particle Size Analysis of Liposomes

The particle size distribution of liposomes was determined by photon correlation spectroscopy (NICOMP Submicron particle sizer, model 370, Nicomp Particle Sizing system, Santa Barbara, USA). All analyses were run in the vesicle mode and intensity distribution (NICOMP). Preparation of samples was conducted in laminar air flow bench using particle free equipment to avoid possible exposure to dust particles. Test tubes were rinsed with sterile filtered medium (acetate buffer, using 0.20 µm pore size filter) prior to dilution of the liposomal suspension in the respective medium until the intensity was within 250-350 Hz. Two cycles with a runtime of 15 min were performed.

### 3.6. Zeta Potential Measurements

Zeta potential measurements were performed on a Malvern Zetasizer Nano ZS (Malvern, Oxford, UK.) The measurement cell was flushed with ethanol and filtrated tap water (0.20 µm pore size filter) before loading of sample. The liposomal suspensions were diluted 1:40 (v/v) with filtered tap water to achieve the optimal measurable concentration. Three cycles for three independent samples of each formulation were performed at 25 °C [53].

### 3.7. Mucoadhesive Properties of Chitosan-Coated Liposomes

The mucoadhesive properties of chitosan-coated liposomes were determined by measuring the in vitro binding of chitosan to pig mucin (PM) [29]. An aliquot of the liposomal suspension (1 mL) was added to 1 mL PM suspension (400 µg/mL in 0.05 M phosphate buffer) and incubated at room temperature for 2 h, followed by centrifugation (Optima LE-80; Beckman Instruments, Palo Alto, CA, USA) at 10 °C with a speed of 216,000 *g* for 1 h. Volumes of 200 µL (four of each sample) were transferred directly from supernatant in the tubes and over to a microtitre plate (Costar^®^ UV 96-well plate with UV transparent flat bottom, Acrylic, Costar^®^, Corning, NY, USA) and the amount of PM was measured spectrophotometrically (Microtitre plate reader; Spectra Max 190 Microplate, Spectrophotometer Molecular devices, Sunnyvale, CA, USA) at 251 nm. The PM binding efficiency was calculated according to Naderkhani et al. [35].

### 3.8. Hydrogel Preparation

Chitosan hydrogels were prepared as previously described [17]. Briefly, medium-molecular-weight chitosan (2.5%, w/w) was dispersed in the blend of glycerol (10% w/w) and acetic acid (0.75% or 1% w/w) and left to swell at room temperature (23–25 °C) for a minimum of 48 h. Hydrogels were diluted in acetate buffer (pH 4.6; comprising 77.1 g/L CH_3_COONH_4_ and 70 mL glacial acetic acid) to obtain hydrogels with the final chitosan concentrations of 0.1% and 0.3% (w/w), respectively. Hydrogels containing acetate buffer or liposomes (20%, w/w) were prepared with the final chitosan concentration of 2.5% (w/w).

### 3.9. Preparation of Biological Fluid Simulants

The preparation of the vaginal fluid simulant (VFS) followed the procedure originally published by Owen and Katz [54]. VFS was composed of 3.5 g/L NaCl, 1.40 g/L KOH, 0.222 g/L Ca(OH)_2_, 0.018g/L bovine serum albumin, 2 g/L lactic acid, 0.16 g/L glycerol, 5.0 g/L glucose, 0.4 g/L urea and 1 g/L acetic acid. The solution was mechanically stirred at room temperature (23–25 °C) to assure a homogenous mixture and the final pH adjusted to 4.5 by addition of 1 M HCl. VFS was stored in the refrigerator (4–6 °C) and was always left for at least 1 h at room temperature prior to experiments.

Semen fluid simulant (SFS) was prepared according to Owen and Katz [55]. In total, four solutions were made separately. Solution 1: 5.24 mL 0.123 M NaH_2_PO_4_ x H_2_O, 49.14 mL 0.123 M Na_2_HPO_4_, 813 mg sodium citrate dehydrate, 90.8 mg KCl, 88.1 mg KOH, 272 mg fructose, 102 mg glucose anhydrase, 62 mg lactic acid, 45 mg urea and 5.04 mg bovine serum albumin. Solution 2: 101 mg CaCl_2_ x 2H_2_O, 15.13 mL H_2_O. Solution 3: 92 mg MgCl_2_ x 6H_2_O, 15.13 mL H_2_O. Solution 4: 34.4 mg ZnCl_2_, 15.13 mL H_2_O. Solution 2 was added slowly into solution 1 under mechanical stirring, followed by solution 3 and 4, respectively. SFS was filtered (0.20 µm pore size filter) and the pH adjusted to 7.7 with 1 M NaOH. SFS was stored in the refrigerator (4–6 °C) and used after being left at room temperature for 1 h.

### 3.10. Determination of Texture Properties of Chitosan Hydrogels

The texture properties of chitosan hydrogels were determined according to the method previously described [17] using Texture Analyzer TA.XT plus (Stable micro systems Ltd., Surrey, UK). The freshly made hydrogels were stored at room temperature prior to the analyses and a 40 mm disc was compressed into the hydrogel (40 g) by the backward extrusion. The test was performed in the compression mode with a pretest speed, test speed and posttest speed of 4 mm/sec. Target mode was set to a distance of 10 mm and all measurements were taken at room temperature (23–25 °C). Five measurements were performed for each hydrogel and the hardness, cohesiveness and adhesiveness of the hydrogels were determined. The measurements were repeated after five days.

### 3.11. Stability 0f Hydrogels in the Presence of Biological Fluid Simulants

Hydrogels were exposed to three different types of biological fluid simulants. Mucin solution alone (PM, 700 µL), a mixture of mucin and vaginal fluid simulant (PM, 635 µL + VFS, 65 µL) and a mixture of mucin, a vaginal fluid simulant and a semen fluid simulant (PM, 570 µL + VFS, 65 µL + SFS, 65 µL) were added onto the surface of freshly made hydrogels and left for 30 min prior to analysis. Texture analysis was performed as described above and five measurements were taken for each sample.

### 3.12. Antibacterial Susceptibility Testing

Chitosan-coated liposomes and hydrogels were assessed for their in vitro antibacterial activity against two clinical species of Gram-positive bacteria, namely *Staphylococcus aureus* and *Staphylococcus epidermidis*, using a modified agar disc-diffusion method [56,57]. Freeze stocks of bacteria isolates were spread on blood agar plates and incubated overnight at 37 °C. A bacterial suspension with a turbidity of 0.5 McFarland was prepared in a saline solution (0.85% w/w). A sterile cotton swab soaked in bacterial suspension was used to draw a cross across the Müller–Hinton agar plates placed on an electrical rotator to achieve uniform plating. Vancomycin (400 µg/mL) was chosen as a positive control due to resistance of chloramphenicol against the bacterial strains. In addition, a negative control was prepared by diluting the non-coated liposomes in same medium as used for coating of liposomes (0.1% w/v acetic acid). Three aliquots (10 µL of samples/controls) were added on the plates, and the plates incubated over night at 37 °C. The inhibition zone was determined by measuring the diameters of the inhibition zones. The antibacterial activity (%) was calculated for each sample based on the inhibition of positive control (100%).

### 3.13. Statistical Evaluation

The student’s t-test was performed to determine the significance of results. The significance level was set to p-value ≤ 0.05.

## 4. Conclusions

We developed chitosan-based mucoadhesive drug delivery systems for treatment of vaginal infections to assure an increased retention time of the drug at the vaginal site and benefit from the antibacterial effects of chitosan. Two mucoadhesive delivery systems were prepared, namely chitosan-coated liposomes and chitosan-based hydrogels. The mucoadhesive properties of chitosan make it a good potential excipient for a vaginal drug delivery system in improving the residence time at the vaginal site. To evaluate the effect of formulation on antibacterial properties of chitosan-based formulations, we challenged formulations against *Staphylococcus epidermidis* and *Staphylococcus aureus* and confirmed chitosan’s antibacterial activity. The antibacterial effect of chitosan appeared to be dependent on the type of bacteria as well as the formulation. Chitosan hydrogels inhibited the growth of both bacteria. The growth of *S. aureus* was only inhibited by 0.3% chitosan-coated liposomes, whereas (0.03%, 0.1% and 0.3%) chitosan-coated liposomes inhibited the growth of *S. epidermidis*. The differences in antibacterial potential need to be further exploited.

## Figures and Tables

**Table 1:** The effect of chitosan coating on liposomal size distribution. The values are presented as the mean ± SD (n = 3).

	Vesicle size				PI*
	Peak 1 (nm)	Weight Intensity (%)	Peak 2 (nm)	Weight Intensity (%)	
Non-coated	226 ± 10.2	89.2	55 ± 4.6	10.8	0.35
0.01	217 ± 0.7	90.2	49 ± 0.2	10.2	0.32
0.03	228 ± 2.4	90.5	54 ± 0.0	10.2	0.31
0.1	288 ± 71.1	74.9	75 ± 19.2	24.6	0.33
0.3	358 ± 90.8	63.5	106 ± 29.4	34.1	0.33

* Polydispersity index.
